# The role of the atopy patch test in the diagnostic work-up of non-IgE gastrointestinal food allergy in children: a systematic review

**DOI:** 10.1007/s00431-023-04994-2

**Published:** 2023-05-30

**Authors:** Barbara Cuomo, Caterina Anania, Enza D’Auria, Fabio Decimo, Giovanni Cosimo Indirli, Enrica Manca, Gian Luigi Marseglia, Violetta Mastrorilli, Valentina Panetta, Angelica Santoro, Marco Ugo Andrea Sartorio, Elisabetta Veronelli, Mauro Calvani

**Affiliations:** 1 Operative Complex Unit of Pediatrics, Belcolle Hospital, 00100 Viterbo, Italy; 2 Department of Maternal Infantile and Urological Sciences, Sapienza University of Rome, 00185 Rome, Italy; 3 Allergy Unit, Department of Pediatrics, Buzzi Children’s Hospital, Milan, 20154 Italy; 4 Department of Woman, Child and General and Specialized Surgery, University of Campania “Luigi Vanvitelli, 80138 Naples, Italy; 5 Pediatric Allergology and Immunology (SIAIP) for Regions Puglia and Basilicata, 73100 Lecce, Italy; 6 Pediatrics Department, Policlinico Riuniti, University Hospital of Foggia, 71122 Foggia, Italy; 7 Pediatrics Department, Pediatric Clinic, Policlinico San Matteo, University of Pavia, 27100 Pavia, Italy; 8 Operative Complex Unit of Pediatrics and Emergency, Giovanni XXIII Hospital, 70056 Bari, Italy; 9 L’altrastatistica srl -Consultancy & Training- Biostatistics office, Rome, Cap 00174 Italy; 10 Mother-Child Department, Pediatric Clinic, University of Parma, 43121 Parma, Italy; 11 Pediatric Allergology Unit, Department of Childhood and Developmental Medicine, Fatebenefratelli-Sacco Hospital, 20121 Milan, Italy; 12 Pediatric Department, Garbagnate Milanese Hospital, ASST Rhodense, 70056 Garbagnate Milanese, Italy; 13 Operative Unit of Pediatrics, S. Camillo-Forlanini Hospital, 00152 Rome, Italy

**Keywords:** Atopy patch test, Food allergy, Non-IgE mediated food-allergy, Motility disorders, Meta-analysis

## Abstract

The “Atopy Patch Test” (APT) has been proposed as a diagnostic tool for food allergies (FA), especially in children with FA-related gastrointestinal symptoms. However, its diagnostic accuracy is debated, and its usefulness is controversial. The aim of this systematic review was to evaluate the APT diagnostic accuracy compared with the diagnostic gold standard, i.e., the oral food challenge (OFC), in children affected by non-IgE mediated gastrointestinal food allergies, including the evaluation in milk allergic subgroup. Both classical non-IgE mediated clinical pictures and food induced motility disorders (FPIMD) were considered. The search was conducted in PubMed and Scopus from January 2000 to June 2022 by two independent researchers. The patient, intervention, comparators, outcome, and study design approach (PICOS) format was used for developing key questions, to address the APT diagnostic accuracy compared with the oral food challenge (OFC). The quality of the studies was assessed by the QUADAS-2 system. The meta‐analysis was performed to calculate the pooled sensitivity, specificity, DOR (diagnostic odds ratio), PLR (positive likelihood ratio), and NLR (negative likelihood ratio) with their 95% confidence intervals (CI). Out of the 457 citations initially identified via the search (196 on PubMed and 261 on Scopus), 37 advanced to full-text screening, and 16 studies were identified to be included in the systematic review. Reference lists from relevant retrievals were searched, and one additional article was added. Finally, 17 studies were included in the systematic review. The analysis showed that APT has a high specificity of 94% (95%CI: 0.88–0.97) in the group of patients affected by FPIMD. Data showed a high pooled specificity of 96% (95% CI: 0.89–0.98) and the highest accuracy of APT in patients affected by cow’s milk allergy (AUC = 0.93).

*      Conclusion*: APT is effective in identifying causative food in children with food-induced motility disorders.

**Table:** 

** What is Known:** *• Atopy patch test could be a useful diagnostic test for diagnosing food allergy, especially in children with food allergy-related gastrointestinal symptoms.*
**What is New:** *• Atopy patch test may be a useful tool in diagnosing non IgE food allergy, especially in children with food-induced gastrointestinal motility disorders and cow's milk allergy.*

** What is Known:**

*• Atopy patch test could be a useful diagnostic test for diagnosing food allergy, especially in children with food allergy-related gastrointestinal symptoms.*

**What is New:**

*• Atopy patch test may be a useful tool in diagnosing non IgE food allergy, especially in children with food-induced gastrointestinal motility disorders and cow's milk allergy.*

## Introduction

Non–IgE mediated gastrointestinal (non-IgE-GI) food-induced allergic disorders encompass numerous and different clinical pictures. Some of these are well-characterized, such as Food Protein-Induced Allergic Proctocolitis (FPIAP), Food Protein-Induced Enterocolitis Syndrome (FPIES), Food Protein-Induced Enteropathy Syndrome (FPE), and Eosinophilic Gastrointestinal Disorders (EGIDs) (including Eosinophilic Esophagitis (EoE), Allergic Eosinophilic Gastroenteritis (AEG), and Eosinophilic Colitis (EC)) [1−3]. Others, especially in the first age of life, may present with less specific symptoms such as acute abdominal discomfort, persistent crying and unsettled behavior, frequent regurgitation or vomiting, and persistent watery diarrhea, often in combination with poor growth or constipation [4]. These latter were recently defined as Food Induced Motility Disorders (FPIMD) [4, 5], meaning all entities not included in the above-mentioned classical non-IgE mediated allergy, which improve after dietary elimination of specific food proteins and which motility alteration has been hypothesized, although the exact pathogenetic mechanisms remain largely unknown [6, 7]. FPMID are included in the group of non-IgE-GI food allergy, as sIgE for foods are not detected in most cases [8].

Diagnosis for non-IgE-GI food allergy is usually based on clinical features by recovery after dietetic therapy and subsequent positivity challenge test. The process is not supported by classical diagnostic tests like skin prick tests (SPT) and serum-specific IgE (sIgE), which are often negative. For these reasons, the APT has been proposed as a tool in the diagnostic work-up. A positive reaction correlates with infiltrating allergen-specific Th2 cells which secrete interleukin 4 and 13 already 24 h after application of the allergen [9], after 48 h a shift towards a Th1 pattern with the secretion of interferon gamma [10] underling the role of APT in delayed reaction type IV, rather than immediate type I reaction.

APT are detected as positive and are mainly useful in delayed/mixed reactions (non-IgE gastrointestinal FA, atopic dermatitis, EoE) rather than IgE mediated FA. However, its diagnostic accuracy remains controversial, and it is not routinely recommended because of the lack of a standardized process and the wide variability in sensitivity and specificity of results in previous studies [11, 12].

Although most studies analyzed APT in groups of patients affected by both immediate and delayed allergic reactions [13-15], recent evidence [16] suggests an increased APT diagnostic efficiency if employed in better-selected cohorts.

Two systematic reviews [17, 18] analyzed the accuracy of APT in patients affected by FA. However, the metanalysis by Luo et al. considered studies including children with different types of food allergies and, in some cases, with atopic dermatitis [17]. The second one [18] provides few informations and does not allow to analyse data from included studies.

The aim of this systematic review was to evaluate the diagnostic accuracy of the APT compared with the diagnostic gold standard, i.e., the oral food challenge (OFC), in children living with non-IgE-GI food allergy, including the evaluation in the milk allergic subgroup.

## Methods

### Search strategy

A comprehensive search was conducted in Medline via PubMed and Scopus (from January 1, 2000, through June 30, 2022), using the keywords “food allergy” and (“patch test” or “atopic patch test”) and (“Food protein-induced enterocolitis syndrome” or “FPIES” or “enterocolitis”), (“Eosinophilic Esophagitis” or “Eosinophilic Colitis” or “Eosinophilic Gastroenteritis”), (“enteropathy” or FPE), (“proctocolitis” or FPIAP), “haematochezia,” “colitis,” “gastritis,” “rectal bleeding,” “failure to thrive,” (“stypsis” or “constipation”).

Two independent researchers (M.U.A.S. and E.M.) screened the databases. The references were imported into a citation manager software (EndNote 20.2.1^®^) for initial duplicate removal.

They independently screened the search string, reviewed all abstracts, and agreed on which full-text articles to retrieve to assess for potentially eligible studies. Disagreements were resolved through discussion, and, if required, in cases of incongruence, a third reviewer (B.C.) was responsible for mediating a discussion and consequent decisions. The systematic review was based on the PRISMA (preferred reported items for systematic reviews and meta-analyses) guidelines, and its protocol was registered in the PROSPERO database. The authors had no conflicts of interest, and the study did not receive any funding.

### Eligibility criteria

We developed a PICOS (patient, intervention, comparators, outcome, and study design) approach to formulate the eligibility criteria for the studies. The following question was set: “Are APT as accurate as OFC for non-IgE-GI food allergy in children?” We consider any kind of OFC, both in open and single- or double-blind form. The studies were not restricted to English-language publications, publication type, or study design; however, they were limited to children (0 to 18 years).

We included only studies that allow us to evaluate the diagnostic performance of APT compared to the gold standard for diagnosing food allergy, i.e., OFC in children affected by FPIAP, FPIES, FPE, EGIDs, or FPIMD. Studies were also included if it could be possible to extract data, and if necessary, additional explanations by contacting the authors were requested.

Studies were excluded if the information was not specific to the topic of this review or if the clinical diagnosis was made without the confirmation by OFC, or if data about specificity and/or sensitivity was not provided or impossible to extract.

### Data collection and analysis

Two authors independently retrieved and reviewed the following data (if available) from all included studies: year of publication, first author, design, population size, mean age, type of symptoms, number of allergic subjects, number of patients with positive sIgE or SPT, APT methods used, and data of its accuracy.

Therefore, eligible studies were classified into two categories: (a) studies considering patients living with classic well-defined clinical pictures like FPIAP, FPIES, FPE, and EDGs; and (b) studies including patients living with FPIMD.

Studies were widely discussed in detail and evaluated by all authors in a standardized and independent manner; the methodological quality was evaluated according to criteria proposed by the Quality Assessment of Diagnostic Accuracy Studies 2 (QUADAS-2) tool [19]. At the same time, any divergence was resolved by discussion and agreement among all reviewers. This instrument judges the risk of bias and accessibility from diagnostic accuracy studies. QUADAS-2 contains four key domains (patient selection, index test, reference standard, and flow and timing), and each domain is rated as low, high, and unclear.

## Statistical analysis

Meta-analysis was performed with the midas command in Stata 16.1. Pooled sensitivity, specificity, positive likelihood ratio (PLR), negative likelihood ratio (NLR), and diagnostic odds ratio (DOR) with their 95% confidence (95%CI) were calculated by a bivariate mixed-effect regression model. The area under the curve (AUC) and relative 95% CI was also calculated. The AUC of summary receiver operating characteristic curve (SROC) results were considered low (0.5 >  = AUC <  = 0.7), moderate (0.7 > AUC <  = 0.9), or high (0.9 > AUC <  = 1). The test could be considered highly informative with PLR exceeding 10.0 and NLR below 0.1; moderately informative with PLR values of 5–10 and NLR of 0.1–0.2; or very lowly informative with LRs of 2–5 and 0.2–0.5. The I^2^ statistic was used to evaluate the heterogeneity between studies; a value of 0% indicates no observed heterogeneity, while values greater than 50% indicate substantial heterogeneity [20].

## Results

**Fig. 1:**
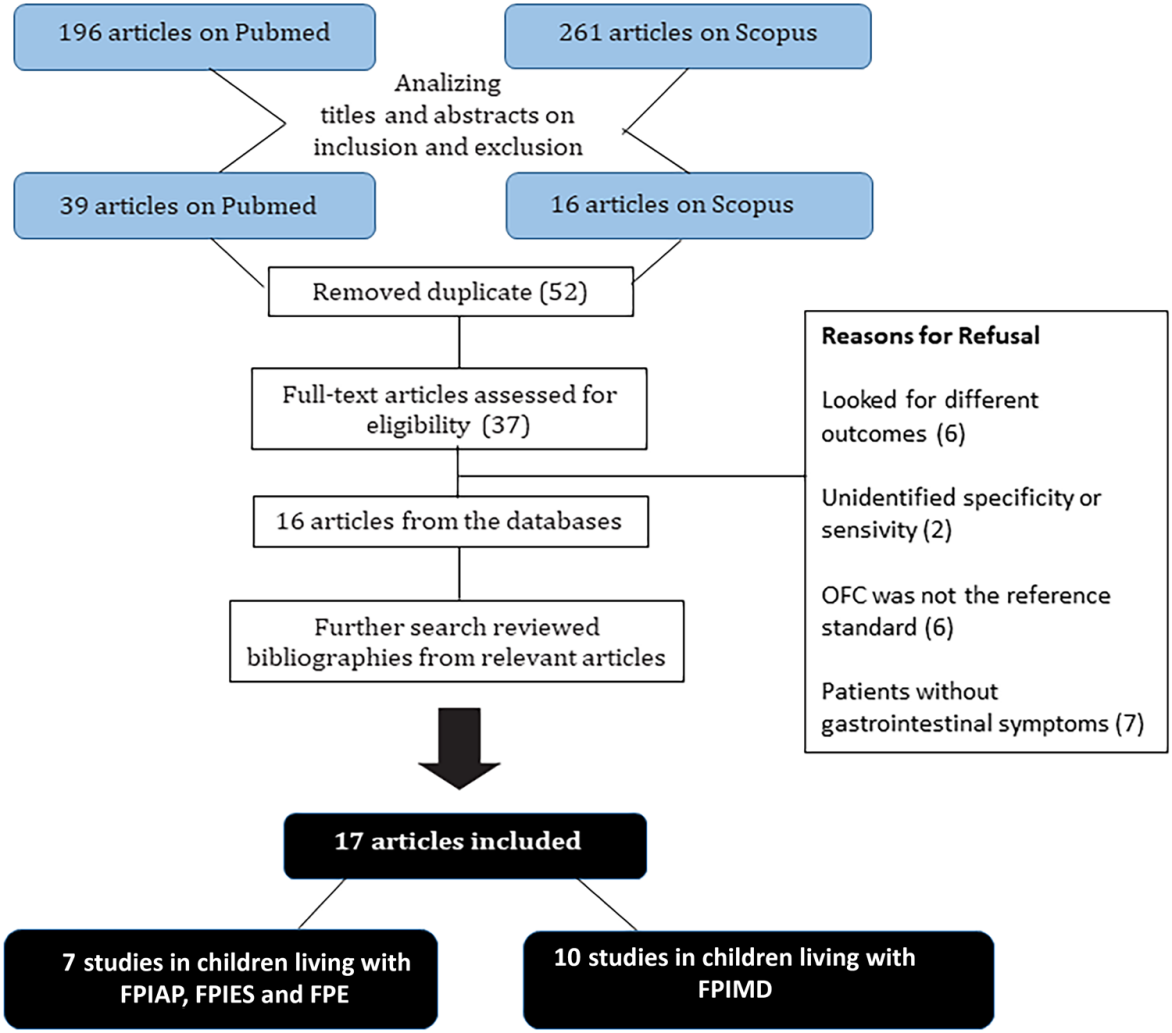
Studies search flow diagram. The electronic databases search identified 196 citations on PubMed and 261 on Scopus. After analyzing titles and abstracts, duplicates were removed and full text were individuated and assessed for eligibility. 16 articles were selected after other ones looked like they had different outcomes or included patients without gastrointestinal symptoms, not comparing to the diagnostic gold standard OFC or not identifying specificity or sensitivity. One additional paper was added from reviewing relevant articles. 17 articles were included in this study

The selection and inclusion process of the studies is reported in the PRISMA Statement Flowchart (Fig. 1). The electronic database search identified 196 citations on PubMed and 261 on Scopus. After analyzing titles and abstracts, respectively, 39 and 16 articles remain. After removing duplicates (3 papers), 52 articles were identified, and the full text was assessed for eligibility. A total of 16 articles were then selected, and the other ones were refused for showing data that did not meet the panned inclusion criteria (Fig. 1). Evaluating the most relevant studies’ references allowed us to detect one additional article. In total, we included 17 studies in this review.

We found three studies evaluating children affected by FPIES [21−23] (Table 1); three studies included patients with FPIAP [24−26] (Table 2), and one included patients affected by FPIAP, FPIES, and FPE [27] (Table 3). Ten studies included [16, 28−36] patients affected by FPIMD (Tables 3 and 4).

**Table 1:**
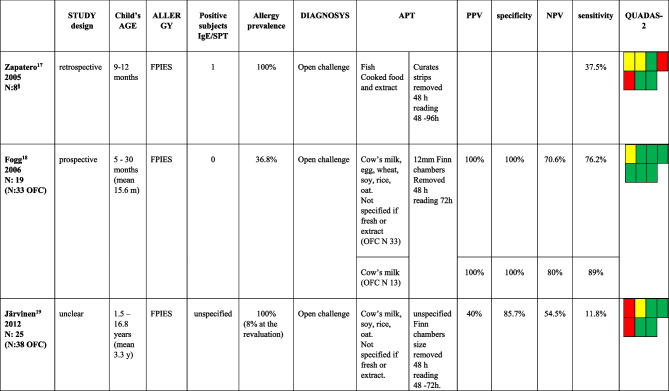
Studies including patients affected by FPIES

*APT* atopy patch test; *PPV* positive predictive value; *NPV* negative predictive value; *OFC* oral food challenge

^§^ data extract from subjects in which APT were performed

**Table 2:**
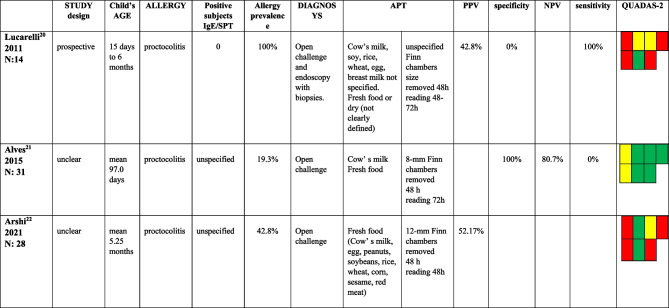
Studies including patients affected by FPIAP

**Table 3:**
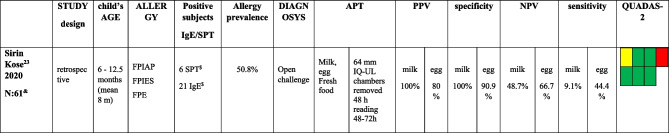
Studies including patients affected by more than one form among FPIAP, FPIES, and FPE

*APT* atopy patch test; *PPV* positive predictive value; *NPV* negative predictive value; *FPIAP* food proteins induced allergic proctocolitis; *FPIES* food proteins induced enterocolitis syndrome; *FPE* food proteins enteropathy

^&^ data extract from subjects affected by GI symptoms

^$^ data from the entire group enrolled, non extracted for those affected by gastrointestinal symptoms

Regarding children living with FPIES, reports were conducted on small patient populations (overall 52 patients) and showed very different results: sensitivity ranged from 11.8 to 89%, while specificity ranged from 85.7 to 100%.

One retrospective study allowed to extract data on only eight patients [21]. Two studies [21, 23] received a lower evaluation on Quadas-2, mainly on those domains concerning patients’ selection. Specifically, the increased risk of bias was based on the exclusive enrollment of patients not representative of the general population as they were already known to be affected by FA when performing OFC and APT. The only prospective study [22] is qualitatively better than the previous and described high values of specificity and PPV (100%) with lower sensitivity and NPV (respectively 76.2% and 70.6%).

Three studies included children with proctocolitis. Based on QUADAS-2, two of them [24, 26] were low-quality studies because the diagnosis were not all confirmed with OFC or apply to a restricted group of selected patients with severe clinical forms not responsive to therapy. The third [25] is qualitatively better than the previous and shows high specificity values (100%) and an NPV of 80.7%.

The only study [27] that enrolled a mixed population affected by FPIAP, FPIES, and FPE is a retrospective one of good quality. It found high values of specificity and PPV for APT performed with fresh foods regardless of which was tested (the best with milk, respectively 100% and 100%, and eggs, 90.9% and 80%). On the contrary, sensitivity values are low (9.1% for milk and 40.4% for egg).

We identified ten studies performed on patients living with FPIMD. These studies have enrolled a large number (n. 770) of children, which were divided into two groups (Tables 4 and 5) to permit separate evaluation of the results of APT diagnostic performance in patients with negative allergy tests and those with mixed positive and negative sIgE-associated forms. Two [28, 30] of these studies allowed data extraction and were included in each group. Thus, three studies included 320 children affected by FPIMD without specific IgE and a negative SPT (Table 4). Nine studies included 598 children affected by FPIMD with or without specific IgE and positive or negative SPT (Table 5). Nocerino’s study [29] was selected for the FPIMD group even if it included a few enterocolitis and enteropathy allergic patients (Table 4).

**Table 4:**
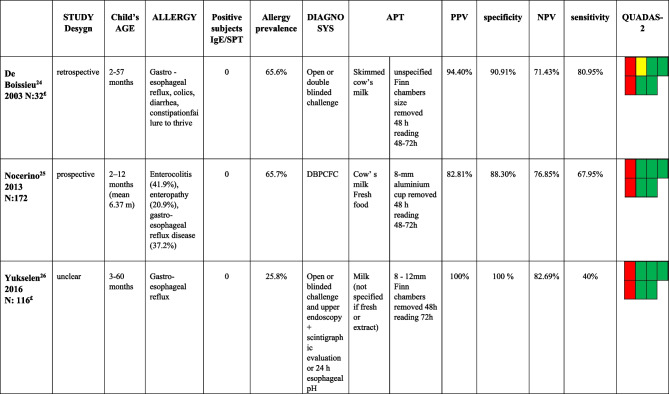
Studies including patients affected by FPIMD without specific IgE and with negative SPT

*APT* atopy patch test; *PPV* positive predictive value; *NPV* negative predictive value; *DBPCFC* double blind placebo controlled food challenge

£ data extract from subjects without specific IgE and with negative SPT

**Table 5:**
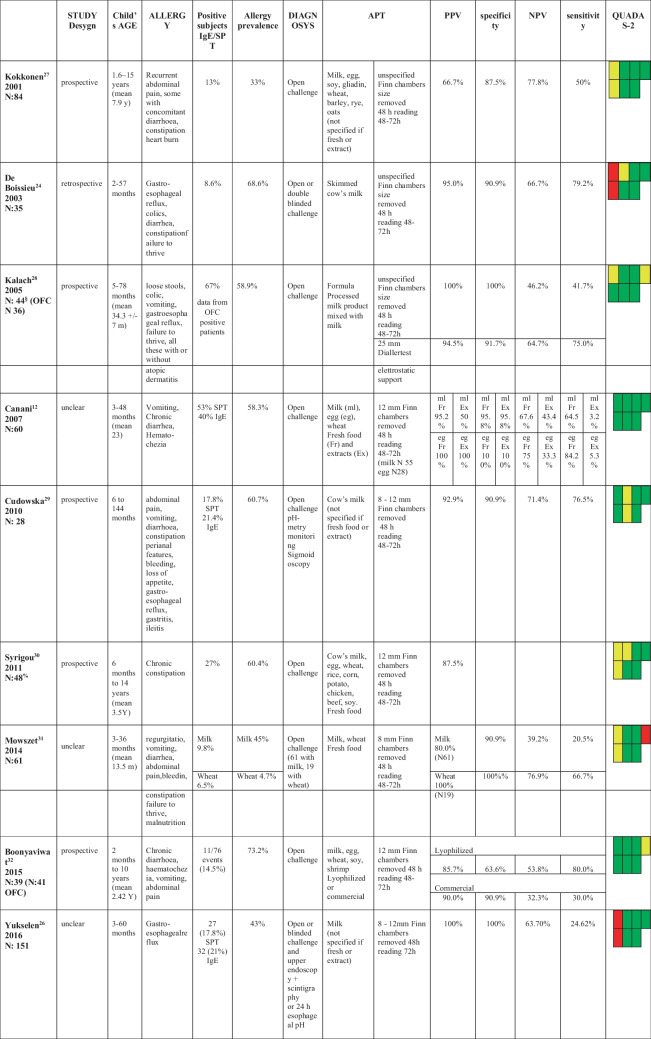
Studies patients affected by FPIMD with or without specific IgE and positive or negative SPT

*APT* atopy patch test; *PPV* positive predictive value; *NPV* negative predictive value

^§^ data extract from children affected by GI-FA

% data extract from children in whom APT were performed

OFC Oral Food Challenge

esophageal pH esophageal pH-metry

Studies that evaluated APT diagnostic accuracy in patients with negative sIgE showed good values of specificity and PPV, respectively, of 88.3–100% and 82.8–100% but poor sensitivity (40–80.9%) (Table 4). However, their quality is poor for enrolling patients not representative of the general population as they were already allergic known persons at the beginning of the study and/or were affected by severe clinical forms.

Data analysis from the second group of FPIMD studies showed that APT has high specificity and PPV regardless of sIgE and/or SPT positivity. In this subgroup, prospective studies received better QUADAS-2 evaluation and reached specificity and PPV values of 95–100% (range values of 63.6–100% and 66.7–100%, respectively).

Canani [16] showed better data when performing APT with fresh food than with food extracts; Alves [25] and Sirin Kose [27] et al. obtained 100% specificity using fresh milk, while most of the other studies do not clearly declare which type of allergenic material had used.

## Methodological quality

### QUADAS-2

**Fig.2:**
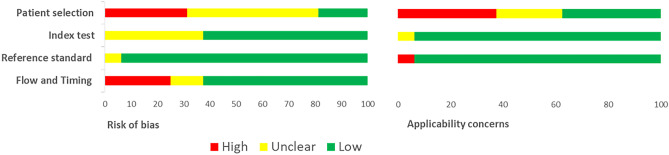
QUADAS-2 results. Proportion of studies with low, high or unclear RISK of BIAS. Proportion of studies with low, high or unclear. Concerns regarding applicability

According to the QUADAS-2 criteria, studies enrolling patients with well-defined gastrointestinal clinical pictures (FPIAP, FPIES, and FPE) and those that included patients affected by non-sIgE FPIMD (Tables 1, 2, 3, and 4) resulted in both in lower judgments on QUADAS-2 evaluation, particularly in the domain dedicated to the selection of enrolled patients (Fig. 2). In most cases, children were not representative of the general population as they were already allergic and/or were affected by severe forms. Since inappropriate exclusions may result in overoptimistic estimates or in underestimation of diagnostic accuracy, the risk of bias was judged as high. The same occurs if APT results were not collected at an appropriate time interval, ideally at the same time as OFC, or if not all the patients receive a diagnosis by positive objective signs at OFC. Studies that enroll subjects with FPIMD both with and without specific IgE (Table 5) appear to be of higher quality.

In general, very few studies have been designed on the specific question of this review [28, 29, 32, 33, 35, 36].

### Meta-analysis results

Due to the small sample size or lack of necessary data in the studies enrolling children affected by FPIAP, FPIES, and FPE, meta-analysis was conducted only in the group of patients with FPIMD. More analysis was also made in the subgroup of children with suspected milk allergy, the most frequent involved food allergen.

For FPIMD, a total of 8 studies analyzed 491 patients. Overall results of the meta-analysis show that APT has high specificity 94% (95% CI: 0.88–0.97) moderate positive likelihood ratio (PLR 8.3 95% CI: 4.1–16.6) and a low negative likelihood ratio (NLR 0.57 95% CI: 0.40–0.82), while sensitivity 46% (95% CI: 0.27–0.66) appears variable between studies. Two out of three studies (Canani et al. and Yukselen et al. [16, 30]) showing lower values for APT were performed with commercial extracts. The heterogeneity is high, with I^2^ always greater than 50%. The AUC value was moderate/high (0.90) with high corresponding DOR of 14 (95% CI: 6–34).

**Fig. 3:**
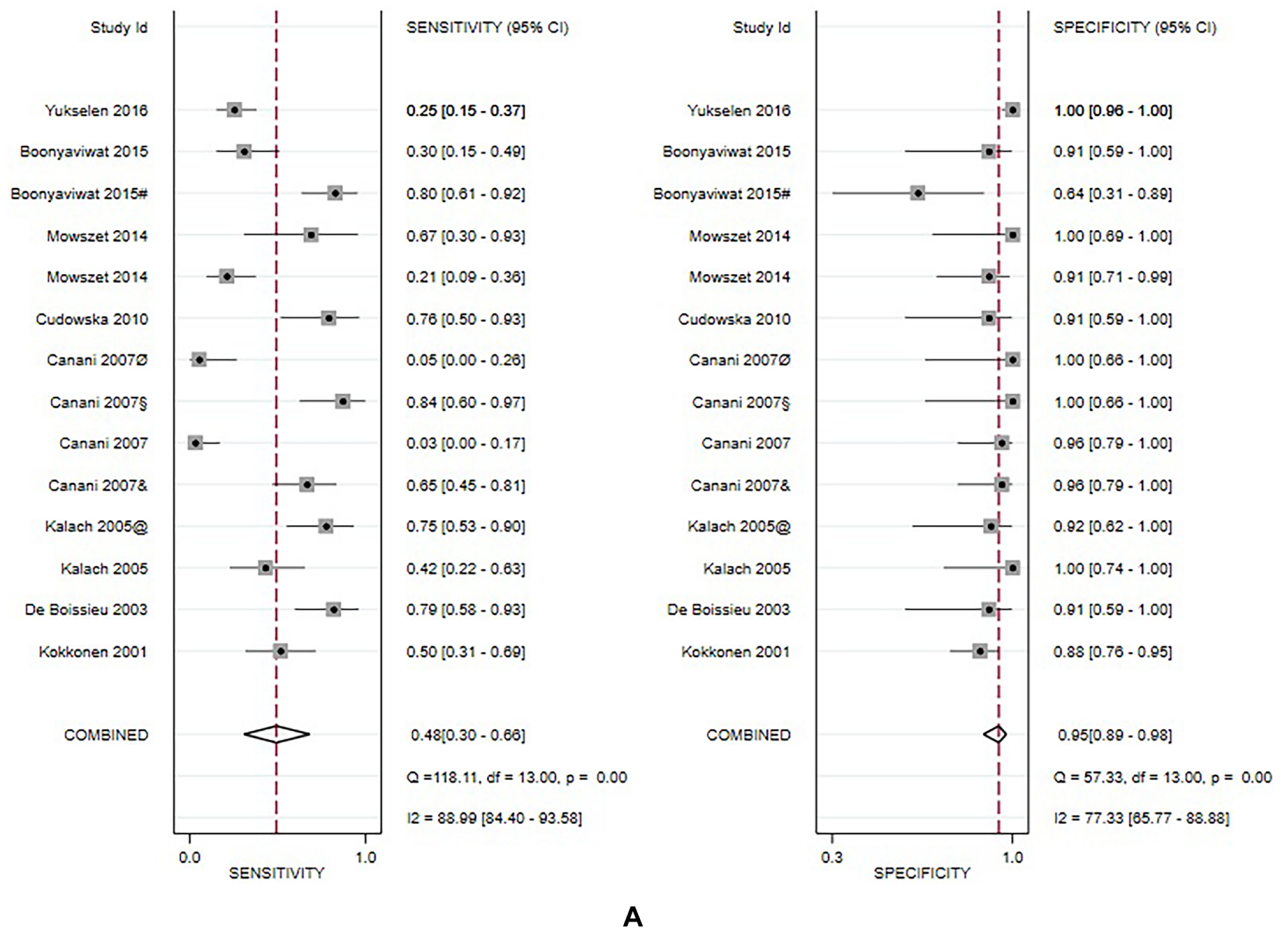

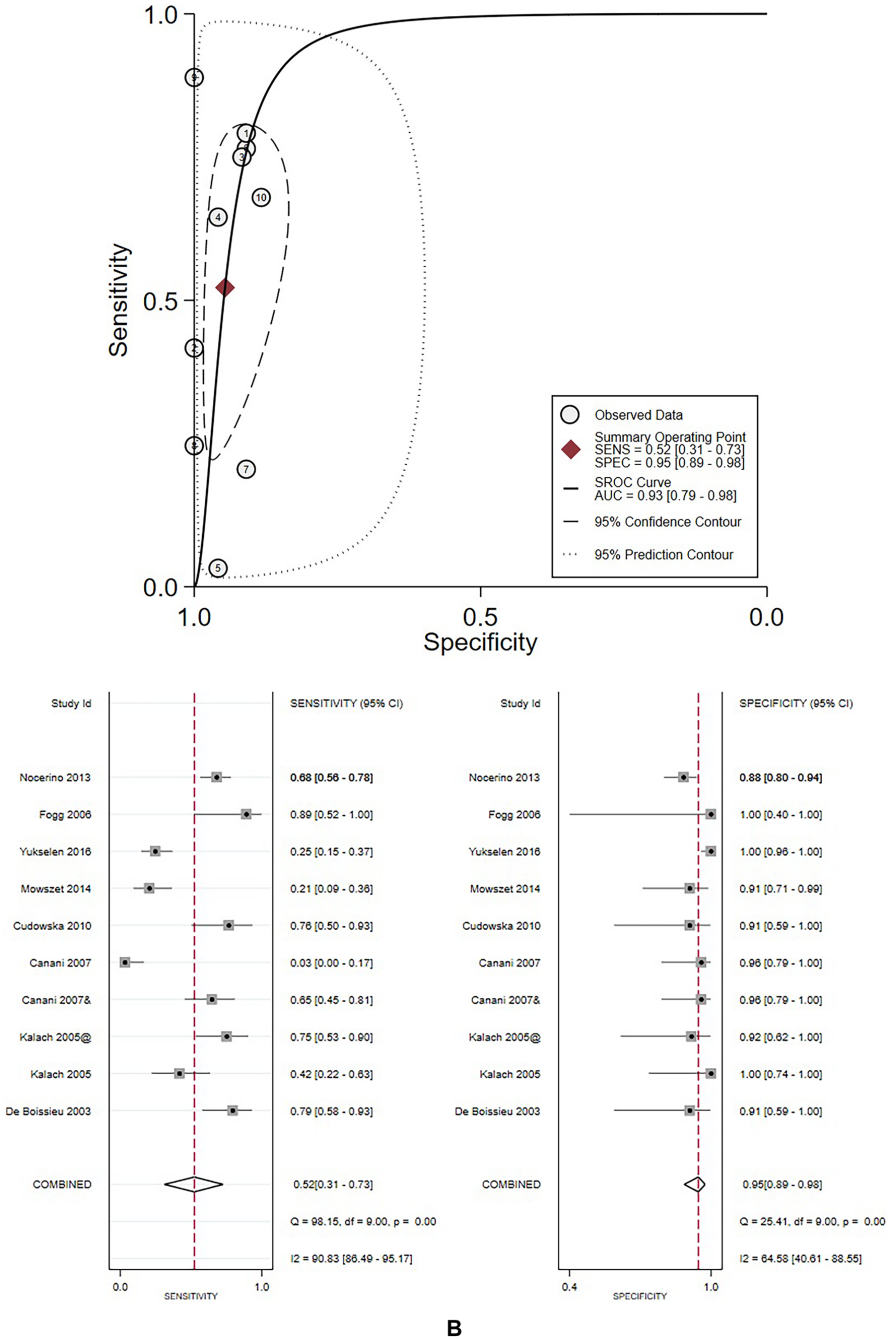
**A** Results of meta-analysis conducted in the group of FPIMD patients. *@ dial, & Fresh, Ø eggs, #Lyophilized,* + *Wheat, § eggs* + *fresh. B Results of meta-analysis conducted in the group of milk allergic patients.* @ dial, & Fresh, Ø eggs, #Lyophilized, + Wheat, § eggs + fresh

The subgroup analysis for milk-allergic patients included eight studies from all clinical groups, including 551 subjects. Seven out of ten studies included FPIMD. Data are like those seen for FPIMD and show even higher pooled specificity of 96% (95% CI: 0.89–0.98) and slightly better accuracy of ATP (AUC = 0.93). The other values are also good: sensitivity 52% (95% CI:0.31–0.73), PLR 9.7 (95% CI: 4.8–19.6), NLR 0.50 (95% CI: 0.32–0.79), and DOR 19 (95% CI: 8–48). Figure 3 illustrates the results of each meta-analysis.

## Discussion

APTs are scarcely used in the diagnosis of FA because their diagnostic accuracy and clinical utility in clinical practice are still debated [11, 12]. The present systematic review aimed to investigate the diagnostic accuracy of the APT compared with the diagnostic gold standard, i.e., the OFC, in children living with non-IgE-GI food allergy. To date, the available data on APT have been obtained in mixed populations of patients suffering from both immediate and non-IgE-mediated FA. A recent systematic review by Luo et al. [17] including 41 studies, aimed to evaluate the diagnostic accuracy of APT in children affected by different clinical pictures of FA with or without atopic dermatitis. In a group sub analysis, they found that APT is specific in children with FA‐related gastrointestinal symptoms. In contrast to the metanalysis by Luo, we aimed to evaluate the diagnostic accuracy of APT exclusively in children with non-IgE mediated food allergy with gastrointestinal symptoms, confirmed by the OFC.

Most available studies focused on FPIMD [8, 37−39] which have not been specifically considered in the previous meta-analysis by Luo et al.

In this group, statistical analysis showed high diagnostic performance of APT, especially in the subgroup of milk-allergic patients (94% with 95% CI: 0.88–0.97, for milk-allergic patients 96% with 95% CI: 0.89–0.98). Thus, APT could help identify offending foods in allergic patients, leading to simplifying the diagnostic process. In fact, in non-IgE-GI food allergy, except for acute FPIES, the delay between food assumption and reaction makes it difficult to suspect the responsible food, while the shortness between food assumption and reaction facilitates the offending food identification in IgE-mediated allergies.

Our search found only a few studies evaluating APT efficacy in the well-known characterized picture of gastrointestinal allergy like in FPIAP, FPIES, and FPE. In detail, studies investigating the APT diagnostic role in FPIES enrolled only a small population and showed different results, probably due to different methodological accuracy. However, the most methodologically correct study by Fogg [22] showed an optimal (100%) specificity and a lousy (76.2%) sensitivity of APT.

Studies enrolling patients suffering from FPIAP also showed very different results. The prospective one obtained a low QUADAS-2 score, and its results contrast with the retrospective one, which was methodologically accurate: the first showed a 100% sensitivity, the second a 100% specificity, and a third unspecified study provided only the low value of VPP (52.17%).

Sirine Kose [27] study enrolled patients affected by FPIES, FPIAP, and FPE and showed that specificity and PPV values are high and varied according to the offending food, both 100% for cow’s milk and 90.9% and 80%, respectively, for hen’s egg. Different food could also account for the different results in the studies. Thus, no definitive conclusion can be drawn about APT role in patients affected by FPIAP, FPIES, and FPE, and meta-analysis was not possible. Further methodologically adequate studies are needed.

In summary, our systematic review provides further important information compared to the two previous meta-analyses. It has been designed on the specific outcome and the search included 4 more studies. Two of those were released later [26, 27]. The remaining two are studies that have been excluded from the previous meta-analysis. We have decided to include them for different reasons. For Kalach’s study [32], we justified its inclusion by extracting data for GI allergy excluding atopic dermatitis. Shirogoy’s study [34] was included after counting non-responder patients to an elimination diet in the group of non-allergic subjects. Our study provides more in-depth results based on the different clinical pictures of GI-FA.

In the FPIMD group for APT in general and for cows’ milk APT, specificity is higher in our study (0.94 and 0.96) than in Luo’s meta-analysis [17] (0.91 and 0.86), respectively. Thus, our data showing a very high AUC in FPIMD and even more for milk allergy indicate that APT is an accurate tool for diagnosing FA in FPMID, especially in the case of CMA. Instead, because of the low sensitivity value, negative APT results cannot exclude an FA diagnosis. For this reason, to increase the APT diagnostic performance, it has been suggested that it could be useful to perform APT joined to the search for sIgE or SPT [16, 36, 40−42].

We did not include studies about APT efficacy in patients affected by EGDIs. These studies were excluded because they did not report sensitivity and specificity [43, 44] or for the absence of comparison between APT results with OFC [45−47]. Most of the studies enrolling patients living with EoE focus on APT efficacy versus exclusion of the suspected food, and diagnosis is generally confirmed on symptom relief and histologic remission without documentation of clinical and histological relapsing after reintroduction of the offending food. Only in Spergel et al. [48] study, OFC was performed but sensitivity and specificity cannot be calculated.

Limits of our systematic review are that our analysis does not allow us to draw any conclusion regarding diagnostic test performance when carried out with fresh, lyophilized, or commercial extracts allergenic foods, as the majority of the studies did not give any data about. Following the analysis, one characteristic that affected our review was the limitation of heterogeneity between studies, which may be explained by non-standardized performing test.

## Conclusions

This systematic review suggests that the APT test may be a useful tool in children living with FPIMD, especially in children affected by CMA.

## Data Availability

**PROSPERO2022** CRD42022322897 Available from: https://www.crd.york.ac.uk/prospero/display_record.php?ID=CRD42022322897

## Abbreviations

AEG: Allergic eosinophilic gastroenteritis
APT: Atopy patch test
AUC: Area under the curve
CI: Confidence intervals
DOR: Diagnostic odds ratio
EoE: Eosinophilic esophagitis
EC: Eosinophilic colitis
EGIDs: Eosinophilic gastrointestinal disorders
FA: Food allergies
FPIAP: Protein-induced allergic proctocolitis
FPE: Food protein-induced enteropathy
FPIMD: Food induced motility disorders
FPIES: Food protein-induced enterocolitis syndrome
LR: Likelihood ratio
NLR: Negative likelihood ratio
non-IgE-GI food allergy: Non–IgEmediatedgastrointestinal food allergy
NPV: Negative predictive value
OFC: Oral food challenge
PICOS: Patient intervention comparators outcome and study design
PLR: Positive likelihood ratio
PPV: Positive predictive value
QUADAS-2: Quality assessment of diagnostic accuracy studies 2
sIgE: Specific IgE
SPT: Skin prick tests
SROC: Summary receiver operating characteristic curve

## References

[1] Calvani M, Anania C, Cuomo B, D'Auria E, Decimo F, Indirli GC, Marseglia G, Mastrorilli V, Sartorio MUA, Santoro A et al (2021) Non-IgE- or Mixed IgE/Non-IgEMediated gastrointestinal food allergies in the first years of life: old and new tools for diagnosis. Nutrients 13(1):226. 10.3390/nu13010226 10.3390/nu13010226 PMC7829867 33466746

[2] Sicherer SH, Sampson HA (2018). Food allergy, a review and update on epidemiology, pathogenesis, diagnosis, prevention, and management. J Allergy Clin Immunol.

[3] Calvani M, Anania C, Bianchi A, D'Auria E, Cardinale F, Votto M, Duse M, Manti S, Tosca MA, Cardinale F, et al (2021) Update on food protein-induced-entero-colitis syndrome (FPIES). Acta Biomed 92: 1–16. 10.23750/abm.v91i11-S.10316 10.23750/abm.v92iS7.12394 PMC9431892 34842596

[4] Canani BR, Caffarelli C, Calvani M, Martelli A, Carucci L, Cozzolino T, Alvisi P, Agostoni C, Lionetti P, Marseglia GL (2022). Diagnostic therapeutic care pathway for pediatric food allergies and intolerances in Italy: a joint position paper by the Italian Society for Pediatric Gastroenterology Hepatology and Nutrition (SIGENP) and the Italian Society for Pediatric Allergy and Immunology (SIAIP). Ital J Pediatrics.

[5] Meyer R, Lozinsky AC, Fleischer DM, Vieira MC, Du Toit G, Vandenplas I, Dupont C, Knibb R, Uysal P, Cavkaytar O (2020). Diagnosis and management of Non-IgE gastrointestinal allergies in breastfed infants - an EAACI position paper. Allergy.

[6] Pensabene L, Salvatore S, D’Auria E, Parisi F, Concolino D, Borrelli O,Thapar N, Staiano A, Vandenplas Y, Saps M (2018) Cow’s milk protein allergy in infancy: a risk factor for functional gastrointestinal disorders in children? Nutrients10–1716. 10.3390/nu10111716 10.3390/nu10111716 PMC6265683 30423934

[7] Schäppi MG, Borrelli O, Knafelz D, Williams S, Smith VV, Milla PJ, Lindley KJ (2008). Mast cell–nerve interactions in children with functional dyspepsia. J Pediatr Gastroenterol Nutr.

[8] Labrosse R, Graham F, Caubet JC (2020). Non-IgE mediated gastrointestinal food allergies in children: an update. Nutrients.

[9] Walter A, Seegräber M (2019). Wollenberg A (2018) Food-related contact dermatitis, contact urticaria, and atopy patch test with food. Nature Clin Rev Allergy Immunol.

[10] Wollenberg A, Vogel S (2013). Patch testing for noncontact dermatitis: the atopy patch test for food and inhalants. Curr Allergy Asthma RepOct.

[11] Muraro A, Werfel T, Hoffmann-Sommergruber K, Roberts G, Beyer K, Bindslev-Jensen C, Cardona V, Dubois A, du Toit G, Eigenmann P (2014). EAACI food allergy and ana-phylaxis guidelines: diagnosis and management of food allergy. Allergy.

[12] Nowak-Wegrzyn A, Chehade M, Groetch ME, Spergel JM, Wood RA, Allen K, Atkins D, Bahna S, Barad A, Berin C (2017). International consensus guidelines for the diagnosis and management of food protein–induced enterocolitis syndrome: executive summary- Workgroup Report of the Adverse Reactions to Foods Committee, American Academy of Allergy, Asthma & Immunology. J Allergy Clin Immunol.

[13] Devillers AC, de Waard-van der Spek FB, Mulder PG, Oranje AP,  (2009). Delayed- and immediate-type reactions in the atopy patch test with food allergens in young children with atopic dermatitis. Pediatr Allergy Immunol.

[14] Pustisek N, Jaklin-Kekez A, Frkanec R, Sikanić-Dugić N, Misak Z, Jadresin O, Kolacek S (2010). Our experiences with the use of atopy patch test in the diagnosis of cow’s milk hypersensitivity. Acta DermatoVenerol Croat.

[15] Mehl A, Rolinck-Werninghaus C, Staden U, Verstege A, Wahn U, Beyer K, Niggemann B (2006). The atopy patch test in the diagnostic workup of suspected food-related symptoms in children. J Allergy Clin Immunol.

[16] Canani RB, Ruotolo S, Auricchio L, Caldore M, Porcaro F, Manguso F, Terrin G, Troncone R (2007). Diagnostic accuracy of the atopy patch test in children with food allergy-related gastrointestinal symptoms. Allergy.

[17] Luo Y, Zhang G-Q, Li Z-Y (2019). The diagnostic value of APT for food allergy in children: a systematic review and meta-analysis. Pediatr Allergy Immunol.

[18] Gayam S, Zinn Z, Chelliah M, Teng J (2018). Patch testing in gastrointestinal diseases-a systematic review of the patch test and atopypatch test. J Eur Acad Dermatol Venereol.

[19] Whiting PF, Rutjes AW, Westwood ME, Mallett S, Deeks JJ, Reitsma JB, Leeflang MM, Sterne JA, Bossuyt PM (2011) QUADAS-2: a revised tool for the quality assessment of diagnostic accuracy studies. Ann Intern Med. Oct 18;155(8):529–36. 10.7326/0003-4819-155-8-201110180-00009. 10.7326/0003-4819-155-8-201110180-00009 22007046

[20] Higgins JP, Thompson SG, Deeks JJ, Altman DG (2003). Measuring inconsistency in meta-analyses. BMJ.

[21] Zapatero RL, Alonso LE, Martín FE, Martínez MMI (2005). Food-protein-induced enterocolitis syndrome caused by fish. Allergol Immunopathol (Madr).

[22] Fogg MI, Brown-Whitehorn TA, Pawlowski NA, Spergel JM (2006). Atopy patch test for the diagnosis of food protein-induced enterocolitis syndrome. Pediatr Allergy Immunol.

[23] Järvinen KM, Caubet JC, Sickles L, Ford LS, Sampson HA, Nowak-WęgrzynA,  (2012). Poor utility of atopy patch test in predicting tolerance development in food protein-induced enterocolitis syndrome. Ann Allergy Asthma Immunol.

[24] Lucarelli S, Di Nardo G, Lastrucci G, D'Alfonso Y, Marcheggiano A, Federici T, Frediani S, Frediani T, Cucchiara S (2011). Allergic proctocolitis refractory to maternal hypoallergenic diet in exclusively breast-fed infants: a clinical observation. BMC GastroenterolJul.

[25] Alves FA, Cheik MFA, De Nápolis ACR, Rezende ÉRMDA, Barros CP, Segundo GRS (2015). Poor utility of the atopy patch test in infants with fresh rectal bleeding. Ann Allergy Asthma Immunol.

[26] Arshi S, Khoshmirsafa M, Khalife M, Nabavi M, Bemanian MH, Shokri S, Seif F, Yousefi A, Fallahpour M (2021) Atopy patch test in the diagnosis of food allergens in infants with allergic proctocolitis compared with elimination/introduction C. Iran J Allergy Asthma Immunol Sep 28;20(5):520–524. 10.18502/ijaai.v20i5.7402 10.18502/ijaai.v20i5.7402 34664811

[27] Sirin Kose S, Asilsoy S, Tezcan D, Atakul G, Al S, Atay O, Kangalli Boyacioglu O, Kangalli Boyacioglu O, Uzuner N, Anal O (2020). Atopy patch test in children with cow’s milk and hen’s egg allergy: do clinical symptoms matter?. Allergol Immunopathol (Madr).

[28] De Boissieu D, Waguet JC, Dupont C (2003). The atopy patch tests for detection of cow's milk allergy with digestive symptoms. J Pediatr.

[29] Nocerino R, Granata V, Di Costanzo M, Pezzella V, Leone L, Passariello A, Troncone TR, Berni Canani R (2013). Atopy patch tests are useful to predict oral tolerance in children with gastrointestinal symptoms related to non-IgE mediated cow’s milk allergy. Allergy.

[30] Yukselen A, Celtik C (2016). Food allergy in children with refractory gastroesophageal reflux disease. Pediatr Int.

[31] Kokkonen J, Ruuska T, Karttunen TJ, Niinimäki A (2001). Mucosal pathology of the foregut associated with food allergy and recurrent abdominal pains in children. Acta Paediatr.

[32] Kalach N, Soulaines P, de Boissieu D, Dupont C (2005). A pilot study of the usefulness and safety of a ready-to-use atopy patch test (Diallertest) versus a comparator (Finn Chamber) during cow’s milk allergy in children. J Allergy Clin Immunol.

[33] Cudowska B, Kaczmarski M (2010). Atopy patch test in the diagnosis of food allergy in children with gastrointestinal symptoms. Adv Med Sci.

[34] Syrigou EI, Pitsios C, Panagiotou I, Chouliaras G, Kitsiou S, Kanariou M, Roma-Giannikou E (2011). Food allergy-related paediatric constipation: the usefulness of atopy patch test. Eur J Pediatr.

[35] Mowszet K, Matusiewicz K, Iwańczak B (2014) Value of the atopy patch test in the diagnosis of food allergy in children with gastrointestinal symptoms. Adv Clin Exp Med 23(3):403–9. 10.17219/acem/37136 10.17219/acem/37136 24979512

[36] Boonyaviwat O, Pacharn P, Jirapongsananuruk O, Vichyanond P, Visitsunthorn N (2015). Role of atopy patch test for diagnosis of food allergy- related gastrointestinal symptoms in children. Pediatr Allergy Immunol.

[37] Ruffner MA, Spergel JM (2016). Non-IgE mediated food allergy syndrome. Ann Allergy Asthma Immunol.

[38] Cianferoni A (2020). Non-IgE mediated food allergy. Curr Pediatr Rev.

[39] Nowak-Wegrzyn A, Mehr KY, SS, Koletzko S,  (2015). Non-IgE mediated gastrointestinal food allergy. J Allergy Clin Immunol.

[40] Isolauri E, Turjanmaa K (1996). Combined skin prick and patch testing enhances identification of food allergy in infants with atopic dermatitis. J Allergy Clin Immunol.

[41] Roehr C, Reibel S, Ziegert M, Sommerfeld C, Wahn U, Niggemann B (2001). Atopy patch test together with level of specific IgE reduces the need for oral food challenges in children with atopic dermatitis. J Allergy Clin Immunol.

[42] Niggemann B, Reibel S, Wahn U (2000). The atopy patch test (APT) a useful tool for the diagnosis of food allergy in children with atopic dermatitis. Allergy.

[43] Erwin EA, James HR, Gutekunst HM, Russo JM, Kelleher KJ, Platts-Mills TA (2010) Serum IgE measurement and detection of food allergy in pediatric patients with eosinophilic esophagitis. Ann Allergy Asthma Immunol 104(6):496–502. 10.1016/j.anai.2010.03.018 10.1016/j.anai.2010.03.018 PMC2963583 20568382

[44] Dalby K, Nielsen RG, Kruse-Andersen S, Fenger C, Bindslev-Jensen C, Ljungberg S, Larsen K, Walsted AM, Husby S (2010). Eosinophilic oesophagitis in infants and children in the region of southern Denmark: a prospective study of prevalence and clinical presentation. Pediatr Gastroenterol Nutr.

[45] Syrigou E, Angelakopoulou A, Zande M, Panagiotou I, Roma E, Pitsios C (2015). Allergy-test-driven elimination diet is useful in children with eosinophilic esophagitis, regardless of the severity of symptoms. Pediatr Allergy Immunol.

[46] Spergel JM, Brown-Whitehorn TF, Cianferoni A, Shuker M, Wang ML, Verma R, Liacouras CA (2012). Identification of causative foods in children with eosinophilic esophagitis treated with an elimination diet. J Allergy Clin Immunol.

[47] Spergel JM, Andrews T, Brown-Whitehorn TF, Beausoleil JL, Liacouras CA (2005). Treatment of eosinophilic esophagitis with specific food elimination diet directed by a combination of skin prick and patch tests. Ann Allergy Asthma Immunol.

[48] Spergel JM, Brown-Whitehorn T, Beausoleil JL, Shuker M, Liacouras CA (2007). Predictive values for skin prick test and atopy patch test for eosinophilic esophagitis. J Allergy Clin Immunol.

